# Correlates of adherence to the Mediterranean diet among preschool-age and school-age children living in Mediterranean countries: a systematic review

**DOI:** 10.1007/s00394-025-03769-9

**Published:** 2025-08-21

**Authors:** Nancy Trezia, Cecile Obeid, Maya Abou Jaoude, Jessica Gubbels, Clara Mazloum, Antoine Aoun, Jessy El Hayek Fares

**Affiliations:** 1 Faculty of Nursing and Health Sciences, Notre Dame University-Louaize (NDU), Zouk Mosbeh, Lebanon; 2 Department of Health Promotion, NUTRIM School of Nutrition and Translational Research in Metabolism, Maastricht University Medical Centre, PO Box 616, 6200 MD Maastricht, The Netherlands; 3 Center for Obesity Prevention Treatment Education and Research (COPTER), Notre Dame University-Louaize (NDU), Zouk Mosbeh, Lebanon

**Keywords:** Adherence, Children, Correlates, Mediterranean diet

## Abstract

**Purpose:**

In recent years, children in Mediterranean countries have shown low adherence to Mediterranean Diet (MD) with a shift toward a Western diet. This study aims to understand the reasons behind moving away from the MD among children, by providing an overview of the different correlates influencing MD adherence among preschool and school-age children (3–8 years old).

**Methods:**

A systematic review was conducted and reported in accordance with the preferred reporting items for systematic reviews and meta-analyses (PRISMA) guidelines and registered in the Prospero database (CRD42023370607). Literature was searched in PubMed, Web of Science and PsycINFO databases for studies, without restrictions on publication date. Inclusion criteria were preschool and school-aged children, studies conducted in Mediterranean countries, and statistical analyses of the association between the different correlates and MD adherence quantified by a validated dietary assessment. Studies that did not provide sub-analyses for the specified age group were excluded. A quality analysis of the included studies was performed using the National Collaborating Centre for Methods and Tools (NCCMT) scale.

**Results:**

A total of 12 studies were included, primarily from European Mediterranean countries, with fewer studies from the Middle East and North Africa. The vast majority of the included studies reported on interpersonal correlates related to the parent’s influence on MD adherence, while child’s age and sex, physical activity, and time spent on video games were classified as individual, and only the place of residence was classified as an environmental correlate. The quality assessment generally showed weak scores.

**Conclusion:**

The primary factors influencing MD were interpersonal correlates related to parents, indicating that interventions should target both children and parents to effectively reinforce MD adherence.

**Supplementary Information:**

The online version contains supplementary material available at 10.1007/s00394-025-03769-9.

## Introduction

The term Mediterranean diet (MD) is often used to describe the typical eating patterns of individuals living near the Mediterranean Sea, including countries such as Greece, Italy, Croatia, Spain, and parts of the Middle East [1]. The MD is characterized by the high consumption of fruits, vegetables, whole grain cereals, legumes, nuts, and seeds, moderate intake of dairy products (milk, yogurt, cheeses), low to moderate intake of fish and poultry, and low consumption of red meat [2]. The ratio of monounsaturated fatty acids to saturated fatty acids is high due to the consumption of olive oil as the main source of dietary fat in the diet, and it is characterized by the prominence of antioxidants, dietary fibers, and vegetable proteins [3].

Studies conducted in Mediterranean countries have shown lower rates of chronic disease and morbidity, and higher life expectancy among adults, associated with the MD [4]. Research on children and adolescents indicates that high adherence to the MD is associated with a healthier metabolic profile [5], lower risk of developing obesity [6], asthma [7], and the diet has anti-inflammatory properties [8]. Further, adherence to the MD among children is associated with better academic accomplishments [9], improved motivation and learning methods [10], and higher life satisfaction [11].

However, over the past decades, a decrease in adherence to MD in Mediterranean countries has been reported. A transition to a so-called Western dietary pattern occurred among different age groups, ranging from preschoolers to adults, characterized by a high intake of processed foods, refined grains, high sugar drinks, and low intake of fruits and vegetables [12, 13]. As a result, 21% of children residing in the Mediterranean countries show low adherence to MD, with only 10% reporting higher adherence scores [8]. Therefore, it is crucial to understand the reasons behind moving away from the MD among children. The number of studies exploring the correlates of MD in children in the Mediterranean region is increasing and have reported multiple factors including individual [14], interpersonal [15], and environmental factors, such as industrialization and modernization [16]. However, no previous systematic reviews have systematically assessed those correlates of adherence among preschool (3–5 years) and school-age (6–8 years) children [17]. Therefore, the current systematic review aims to assess the correlates of adherence to the MD among this age group (3–8 years old), using the socio-ecological model (SEM) [18]. The SEM framework includes intrapersonal, interpersonal, and environmental levels of influence, emphasizing the complex multifactorial origin of a certain context [19]. The results of this review will guide efforts aimed at increasing adherence to the MD, identifying the most vulnerable groups with the lowest level of adherence, and informing programs or policies supporting improved adherence to the MD on the correlates that should be addressed.

## Methodology

A systematic review of studies reporting on the correlates of adherence to the MD among preschool and school-age children from Mediterranean countries was conducted and reported in accordance with the PRISMA 2020 guidelines [20]. The review protocol was registered in the Prospero database under the registration number: CRD42023370607.

### Selection criteria for studies

The target population includes healthy preschool and school-aged children between 3- and 8-years old living in a Mediterranean country that traditionally follows a MD (Spain, France, Italy, Croatia, Bosnia, Albania, Greece, Turkey, Syria, Lebanon, Israel, Palestinian territory, Egypt, Libya, Tunisia, Algeria, Morocco, Cyprus, Malta, Monaco, Montenegro, Slovenia, and Gibraltar) [3]. Studies included in the review are quantitative observational (e.g., case–control, prospective cohort, and cross-sectional studies) and experimental (e.g., randomized controlled trials) studies published in the English language, without any further restrictions on the type of study designs. Hence, both cross-sectional and longitudinal studies were eligible to be included. Articles describing original research were included, hence reviews, conference abstracts, etc. were excluded. There were no restrictions on the publication date. Based on the Centers for Disease Control (CDC), preschool and school-age children are defined from 3 to 5 years and 6 to 8 years respectively [17], and thus selected as our target population in the systematic review. The study was excluded if a broader age range was included without conducting a sub-analysis for the age group in question. Articles providing statistical analyses of the association between the different correlates (individual, interpersonal, or environmental), and MD adherence quantified by a validated dietary assessment and scoring tool among the target age group, were retained. Whenever different types of analyses were presented, the results of the most advanced statistical test were extracted (e.g., adjusted for potential covariates). Studies that reported solely on correlates among Mediterranean countries were retained and those done on countries outside the Mediterranean basin were excluded.

### Literature search

A systematic search was conducted in three electronic databases PubMed, Psych INFO, and Web of Science, until April 2024. The search strategy was formed by a combination of controlled descriptors (indexers in each database) and keywords, according to the indication offered in each electronic database. Some of the keywords used are “Mediterranean diet,” “determinants”, “correlates”, “environmental conditions, “and “interpersonal factors”. The complete search strategy for all the databases is highlighted in Supplementary material 1 (S1). The search strategy was the result of various iterations to reach the final one used for this systematic review. All the selected studies were imported to the Rayyan software web application [21]. Duplicates were identified and removed by (NT) and (CM). The remaining articles were divided for initial screening by 2 reviewers (NT) and (CM) on a title and abstract basis. Studies that did not meet all the inclusion criteria were excluded and this was confirmed by three other authors (JH), (MAJ), and (CO). Potentially relevant studies were identified on a full-text basis for inclusion by (NT) and (CM) against the inclusion/exclusion criteria listed prior to the initiation of the search strategy. All included articles were further confirmed by the remaining authors. These processes were performed independently, and a discussion to resolve disagreements was fulfilled in each step with (JH), (MAJ), and (CO) to achieve consensus.

### Data extraction and quality assessment

Two assessors (NT and CM) independently performed the extraction and tabulated data in an Excel file, including title, first author’s name, publication year, study region, study design, data collection date, sample size, population’s characteristics (age range, sex, etc.), instrument for the assessment of MD adherence, mean adherence score, type of correlates, and association between MD adherence score and the various correlates (Table 1). The strength of the reported associations was classified according to Cohen’s standard thresholds for effect sizes, to facilitate interpretation and comparability across studies [22]

**Table 1:** Descriptive characteristics of the included studies

References	Study design (name of cohort)	Date of data collection	Nationality	Sample size (n =)	Sample characteristics	Tool	Mean MD adherence scores	Correlates	Type of statistical test
[26]	CS (Program SI!)	October–November 2011	Spanish	2062	Mean age(SD): 3·73 (0·93) Age Range 3–5 Girls: 49·5% Region: Urban	KIDMED	7.38(2.04)	Parent's Age Parent's Educational Level Parent's Income Parental Healthy Dietary Attitudes, Habits, Knowledge, & Practices	Pearson’s correlation
[31]	CS	March–October 2019	Croatian	598	Mean Age (SD): 5(1) Age range 3–7 Region**:** Urban	KIDMED	NR	Child's Age Child's Sex	Spearman’s rank correlation
[32]	CS	March–April 2017	Croatian	260	Age Range: 5–6 Girls: 48.46%	KIDMED	NR	Child’s Sex Child’s PAL	Spearman’s rank correlation
[24]	CS (The SENDO project)	2015–2017	Spanish	287	Mean Age(SD): 7.2 (1.81) Age Range: 5–7	KIDMED	NR	Parental Healthy Dietary Attitudes, Habits, Knowledge, & Practices	Multivariable linear regression
[28]	CS	2018–2019	Italian	379	Age Range: 6–7 years Girls: 48.7%	KIDMED	NR	Child's PAL Time spent on Video Games (> 1h) Parent's Educational Level Maternal Health Consciousness	Multivariate logistic regression
[33]	CS	October 2017–April 2018	Greek	401	Mean Age (SD): 7.7 (3.1) Age Range: 2–12 Girls 53.8% Region: Urban	KIDMED	7.1(2.4)	Parental Healthy & Unhealthy Dietary Attitudes, Habits, Knowledge, & Practices	Multiple linear regression
[35]	CS	February–June 2019	Turkish	1431	Mean Age (SD): 4.7 (0.7)	KIDMED	6.5(2.1)	Parent's Employment Status Parent's Educational Level Parent's Divorce Parental Healthy & Unhealthy Dietary Attitudes, Habits, Knowledge, and Practices	Pearson’s correlation
[29]	CS	2017–2018	Italian	282	Mean Age (SD): 7.62 ( 0.84) Age Range: 6–8 Girls: 44.3%	KIDMED	4.39(2.30)	Child's Age Child's Sex Child's PAL Parent's Employment Status Parent's Educational Level Place of Residence	ANOVA and Chi square
[34]	CS	September 2018–January 2019	Cypriot	70	Mean Age (SD):3.21 (0.94) Age Range: 2–6 Girls: 44.3%	KIDMED	9.05(2.02)	Child's Sex Parent's Educational Level Mother's Adherence to MD	Chi square and Pearson’s correlation (for mother’s adherence to MD)
[30]	CS	NR	Italian	1164	Mean Age(SD):7.0 (0.9) Age Range: 6–8	IMI	Winter: 3.5 (1.7) Spring: 3.3 (1.7)	Child's Sex Parent's Educational Level Parent's Income	Chi square
[25]	CS	NR	Spanish	363	Age Range: 3–5 Girls: 46% Region: 66% Urban	KIDMED	NR	Child's Age Child's Sex Place of Residence	t-test
[27]	Cohort (The SENDO project)	January 2015–June 2022	Spanish	979	Mean age(SD):5.01 y., (0.85) Boys: 51% Age range: 4–5 years	KIDMED	5.5 in none-breastfed infants 5.7 breastfed < 6 months 6.4 in those breastfed > 6 months	Breastfeeding	Logistic regression

NR not reported

CS cross-sectional, KIDMED Mediterranean Diet Score Tool for children and adolescents, IMI Italian Mediterranean Index, MD Mediterranean Diet, PAL Physical Activity Level

^a^Data reported based on their availability in the articles

Quality assessment was conducted using the National Collaborating Center for Methods and Tools (NCCMT) scale for the assessment of quantitative studies which was deemed most appropriate for the cross-sectional study designs included in this review [23]. The NCCMT tool assesses in total seven domains of study quality: selection bias (representativeness of the sample), study designs (randomization of the sample), confounders, blinding, data collection methods, withdrawals and dropouts, and intervention integrity. For better assessing the studies included, the authors decided to replace the seventh aspect of the tool (concerning intervention), with an evaluation of the validity and reliability of the tools used for exposure assessment. The intervention integrity aspect was removed due to its lack of relevance to the respective research question and objectives of the systematic review. Although all seven aspects were assessed, only four were highlighted as being relevant to the type of studies included in the present review, focusing on providing a cross-sectional description of adherence to the MD meeting the initial objectives. This included an assessment of the following aspects: selection bias, blinding, data collection methods, and validity and reliability of the tool. Each of these aspects was assessed using three categories based on the total score to interpret the certainty of evidence: strong, moderate, and weak. An overall quality score was incorporated into the article in question as follows: strong (no weak ratings), moderate (one weak rating or no weak ratings but mostly moderate ratings), and weak (two or more weak ratings) [23]. To ensure inter-rater reliability, 38.5% of the papers were assessed independently by all four reviewers. A formal inter-rater reliability coefficient was not computed, as the review employed a structured consensus approach during the initial screening phase rather than independent parallel screening required for such statistical calculation. Consensus was reached through discussion for all discrepancies, and a 100% agreement was achieved before proceeding with the independent assessment of the remaining studies.

### Data analyses

Summary tables of the retrieved studies were developed to describe the association between MD adherence in children aged 3–8 years and its potential correlates. The SEM was used to organize the categories of the different correlates: individual, interpersonal, and environmental [18]. Accordingly, correlates extracted from the included studies were classified as corresponding to these three levels: factors related to the children were identified as individual, interpersonal correlates were related to the parent’s influence, and the environmental factors reflected the setting that the child was exposed to. We reported the results of the most advanced statistical analyses (e.g., adjusted for covariates) performed between the different correlates and the MD adherence score. Each association was then classified into one of three categories: a significant positive association, a significant negative association, or no significant association (based on p-values). Further details of this classification are provided in the results section in Table 2.

**Table 2:**
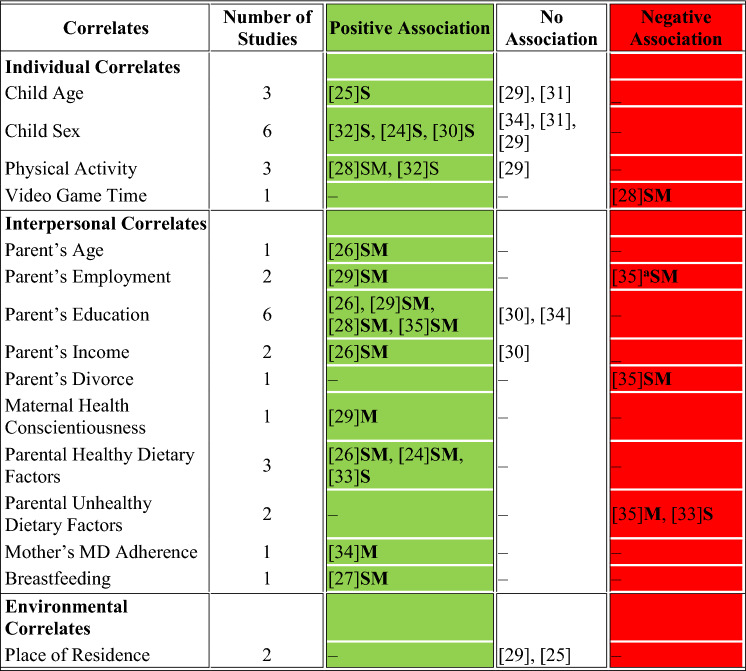
Overview of correlates influencing MD adherence that were found in the included studies

^a^MD adherence was statistically significant with part-time working mothers; Abbreviations of classification of effect sizes: S: small, M: medium

## Results

### Study selection

**Fig. 1:**
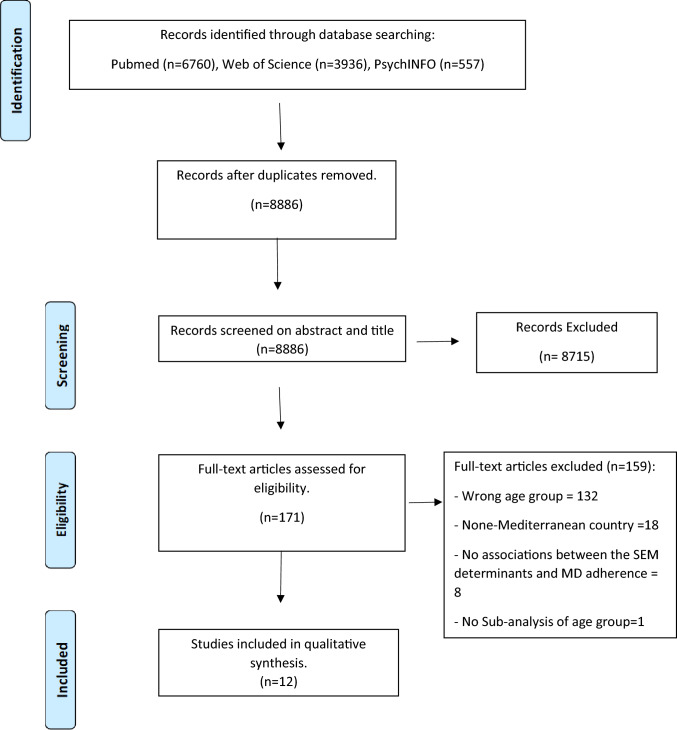
Flow chart result of the search strategy

The search of the 3 databases yielded a total of 11,253 studies: PubMed (n = 6760), Web of Science (n = 3936), and Psych INFO (n = 557). A total of 8886 articles remained after duplicate removal and were eligible to be screened on a title and abstract basis. Of the 8886 articles, 8715 were excluded upon title and abstract screening, and 171 articles remained eligible for full-text screening. Out of these, 159 articles were excluded. Reasons to exclude articles were: the target age group (3–8 years) was not exclusively assessed in the studies or sub-analyses (n = 132), none-Mediterranean country (n = 18), studies that did not conduct any statistical analysis assessment of the associations between the potential SEM correlates and MD adherence (n = 8), and the study included a broader age range without conducting a sub-analysis for the age group in question (n = 1). The final number of included studies for qualitative assessment was n = 12. The result of the selection procedure is summarized in Fig. 1.

### Characteristics of included studies

The included articles involved 11 cross-sectional studies and one cohort study which were published from 2015 until 2023. The sample size in the included studies ranged between 70 and 2062 children. Included studies conducted in Spain (n = 4) formed the largest group [24–27], followed by Italy (n = 3) [28–30], Croatia (n = 2) [31, 32], and one from each of Cyprus, Greece, and Turkey [33–35].

Results on MD adherence were reported using the KIDMED in 11 studies, while only one study used another scoring tool: the Italian Mediterranean Index (IMI) [30]. These tools emphasize the different components of MD and their respective consumption and are known for their high validity and reliability in assessing Mediterranean dietary patterns among children and adolescents [36]. The mean MD adherence score for preschoolers and school-age children was reported in 7 articles and ranged from 3.3 to 9.05 indicating poor to moderate adherence [37]. Several factors were assessed as potential determinants of MD adherence and were across all categories of the SEM. The strength of associations across the included studies ranged from small to medium effects. Overall, most positive associations between individual, interpersonal, and environmental correlates and MD adherence were of small magnitude, with a few moderate effects observed, particularly for parental factors such as maternal health consciousness and parental dietary behaviors (see Table 2).

### Quality analysis of the included studies

Based on the NCCMT rating system, the overall quality assessment showed weak scores among 10 articles while only one article scored moderate and one scored strong [26, 28]. Among the studies that had a weak rating, seven had a low-quality score on the selection bias criterion evaluating the representativeness of the sample toward the general population and regarding whether the study failed to report on the percentage of the population that agreed to participate in the study [24, 25, 27, 28, 30, 34, 35], 2 articles scored moderate [29, 31], and one article had a strong rating [32]. All articles had weaknesses in the confounders section (selection bias) except for two articles that had strong scores [26, 28]. Concerning the validity and reliability of the data collection tools, 5 studies had a moderate score where the collection tools used were valid, but their reliability was not reported [24, 26, 29–31]. The quality assessment showed moderate to strong scores for the data collection methods; 6 studies had moderate scores, 5 had strong, with only one study had a weak score [27]. Regarding the exposure assessment rating, 4 articles were weak, 3 articles had a moderate score and 5 articles were strong [25, 27, 28, 32, 33].

More details about the quality assessment of the included studies are provided in Supplementary Material (S2).

### Correlates of MD adherence

The various correlates reported in the included studies were classified according to the socio-ecological model (SEM): Individual, Interpersonal, and Environmental. Four correlates were classified as individual, another ten were classified as interpersonal and only one was classified as an environmental correlate. More details about MD adherence and the classification of correlates are provided in Table 2.

### Individual correlates

Four individual correlates were identified across the included studies: child age and sex, physical activity level, and time spent on video games [25, 29–32, 34]. Three articles reported on child age, but only one showed a significant positive association [25], while the two remaining articles showed no association. In terms of the child’s sex, 3 out of 6 articles reported a statistically significant higher adherence to MD among boys compared to girls [25, 30, 32], while the remaining articles showed no association. Being physically active was significantly positively associated with the degree of adherence to MD [28, 32]. However, it was significantly negatively associated with the number of hours spent on video games [28].

### Interpersonal correlates

Ten different correlates were classified as interpersonal and were mostly related to parents’ characteristics: parent’s age, employment status, education level, parent’s income, parent’s divorce, maternal health consciousness, parental healthy dietary practices, knowledge and attitudes, alongside unhealthy habits such as parenteral restriction of child’s food intake and using food as a reward, mother’s adherence to MD, and breastfeeding. Only one study reported on parents’ age and showed a positive association, where children whose parents aged 37 years or older had higher MD adherence compared to younger parents [26]. The two studies addressing parental employment yielded different results: one study showed that children of employed parents compared to unemployed mothers, had a significantly higher degree of adherence to MD [29], while the second study showed a significant negative association between part-time working mothers and children’s adherence compared to unemployed mothers [35]. A higher parental education was associated with higher MD adherence among children, and this was significant across 4 articles that assessed it as a potential determinant [26, 28, 29, 35], while no association was seen in the two remaining articles [30, 34]. Parent’s income showed no association with adherence to MD in one study [30], while another reported significantly higher MD adherence with high-income status [26]. Children whose parents were separated had significantly lower MD adherence compared to children whose parents were together [35]. Maternal health conscientiousness and mother’s adherence to MD were both significantly positively associated with higher MD adherence among their children [28, 34]. Three articles reported a statistically positive association of MD adherence with parental healthy eating attitudes, habits, knowledge, and practices [24, 26, 33] while two others showed a significant negative association with unhealthy attitudes and practices in the studies [33, 35]. Further, being breastfed was significantly associated with a higher degree of adherence to a MD in children (n = 1) [27].

### Environmental correlates

Only the place of residence was classified as an environmental determinant. The two articles examining this reported no significant association [25, 29].

## Discussion

This systematic review provided an overview of the correlates of adherence to the MD among preschool and school-aged children living in Mediterranean countries. The search strategy identified 12 articles published in recent years from 2015 until 2023, that fulfilled the eligibility criteria for inclusion and classified the correlates according to the SEM. The majority of the studies had low-quality evidence and were focused on interpersonal correlates.

Most studies classified preschool and school-age children as having a poor-to-moderate adherence to the MD according to the KIDMED test or the IMI [26, 27, 29–32]. In line with this, a previous systematic review also found low to moderate adherence among adults in Mediterranean countries [13]. This raises the question whether children with poor adherence are growing into poor MD-adherent adults or are the low adhering adults of Mediterranean countries influencing their children’s adherence. However, no longitudinal studies have addressed this association, which could be interesting to assess in future research. However, previous studies have shown that childhood dietary habits can influence later adult behavior [38], as well as the substantial influence of adults (particularly parents) on children’s eating habits constitute the basis for shaping long-term dietary habits [38]. This might indicate that, over time, MD countries are trapped in a downward spiral of low-adhering parents raising low-adhering children, who in turn become low-adhering parents themselves, drifting further and further away from the MD. This also highlights the urgent need for actions to improve MD adherence across the lifespan, targeting both children and adults who are moving away from MD. Family and school-based interventions that address the dynamic interplay between parents and children together can be effective in promoting healthy eating habits [39–41].

The studies included in this review, generally reported on correlates of children’s MD adherence in European Mediterranean countries like Spain, Italy, Croatia, Cyprus, Greece, and Turkey, while studies were lacking from other regions in the Mediterranean basin including the Middle Eastern (e.g., Lebanon, Syria) and African (e.g., Algeria, Egypt, Tunisia) Mediterranean region. This highlights the need for more studies on this same age group from the remaining Mediterranean countries to examine potential regional differences. Although the MD presents common characteristics of food components consumed across countries, differences could be encountered from one region to the other in their staple foods [42].

The MD adherence was associated with several individual factors: child age and sex, physical activity level, and time spent playing video games. In terms of sex distribution, the findings highlighted stronger MD adherence in boys compared to girls [29, 34], with only one article reporting significantly stronger adherence among girls [30]. The difference in adherence could be explained by the fact that boys in their preschool years might adopt their mothers as role models, and thus if the mother is more adherent this could be reflected in higher adherence among boys [39], however, this potential explanation is from limited evidence and further studies are needed on this matter. The remaining articles did not examine sex differences in adherences, at this point, it cannot be assumed that sex can affect adherence rate and therefore cannot tailor sex-specific interventions [24–28, 31–33, 35]. Age was also identified as an individual determinant, where it was observed that preschoolers and school-age children had higher degree of MD adherence compared to secondary school. This association was statistically significant in only one study that discussed age as a potential determinant [25]. Perhaps better adult supervision and control among school-aged children could play a role [25]. MD adherence tends to decrease as children get older as shown in [43], probably related to more independence from their parents in terms of food choices. Similarly, another systematic review assessing the adherence to the Mediterranean diet by the Greek and Cypriot population also reported higher adherence among the younger (3–12 years old) compared to the older (13–18 years old) aged children, accentuating the role of parents in increasing children’s adherence to the MD [44]. This finding stresses the importance of early intervention, since behaviors acquired during childhood can persist into adulthood and lead to long-term habits [45].

Thus, this systematic review identified the role of parents as the main interpersonal correlate: parent’s age, employment status, education level, parent’s income, parent’s divorce, maternal health consciousness, parental healthy dietary practices, knowledge and attitudes, alongside unhealthy habits such as parenteral restriction of child’s food intake and using food as a reward, mother’s adherence to MD, and breastfeeding. Most of the studies showed that a higher percentage of optimal MD adherence was observed in children of parents with a higher educational level [26, 28, 29, 35]. Indeed, parents can influence the choices their children make by means of food accessibility and providing healthy eating guidance [24, 26, 46]. Educated parents can have better nutritional knowledge and increase the availability of healthy food items that can be relatively expensive as shown in [47]. Similarly, a systematic review that identified potential correlates of MD adherence in adults found that financial, socio-cultural, as well as food accessibility and availability can collectively affect the scope of adherence [48]. This suggests the similarity of the barriers and facilitators that are associated with the adoption of adherence to MD in both adults and younger age groups.

Similarly to our results, the parental influence on children’s dietary patterns has been widely investigated through significant associations across different studies. A recent systematic review among schoolchildren (7–10 years old) showed that the parent’s eating habits are strong predictors of children’s food practices and preferences since the home environment is the first place where the child receives nutrition education and thus establishes dietary habits​ [49]. Both the parent’s and children’s dietary practices are modifiable risk factors that merit high attention since poor dietary habits established during childhood might persist into adulthood [46, 50, 51]. In addition, parents aged 37 years or older had a better MD adherence and consequently their children, this might be related to the gaining of nutritional knowledge and consciousness with age [26, 52]. Also, children whose parents are separated had a low KIDMED score, this could be attributed to divorce which can affect the home environment by the absence of either parent and place pressure to make additional efforts to compensate for the missing person and gain the child’s affection by providing unhealthy foods and promoting food intake for reasons other than hunger [35, 53]. Furthermore, if mothers are not present all the time, or in the case of shared custody, mothers would have less input in the selection and offering of food [26], and less involvement in culinary activities [54] which may also affect MD adherence. The sole potential environmental determinant examined was place of residence, which was not found to be significantly associated with MD adherence.

While these findings highlight the pivotal role of parental factors, it is worth noting that the strength of these associations was generally small to moderate. This suggests that although parents substantially shape children’s eating behaviors, individual factors alone may exert a limited effect, and broader socio-ecological influences—including cultural norms, school food environments, and community resources—should be considered in future research and interventions aiming to enhance MD adherence among children. [55]

The quality assessment showed weak to moderate scores on selection bias, validity and reliability of measurement instruments, and data collection criteria from the NCCMT scale for assessment of quantitative studies for most of the included studies [22]. This indicates the need for more high-quality studies among Mediterranean countries, especially in terms of the representativeness of the general population and the use of validated and reliable measurement tools. In addition, this systematic review included observational studies only (11 cross-sectional and one cohort study), since the availability of randomized control trials (RCT) was limited among this age group. Therefore, there is a need for longitudinal and experimental studies for higher-quality evidence.

The findings of this review highlight the need for multi-level interventions that address both individual and environmental determinants of MD adherence in early childhood. In the school setting, integrating nutrition education into the curriculum, improving the nutritional quality of school meals, and involving families in healthy eating initiatives could foster healthier dietary patterns [56]. At the policy level, supporting parental education, regulating food marketing to children, and encouraging community-based programs can further promote adherence to the MD from a young age [57–59] These efforts should be culturally tailored and sustained across settings to maximize their impact.

This systematic review has several strengths and limitations. Among the limitations, the search strategy was limited to articles in English, excluding potential further studies written in other languages of the Mediterranean countries (Spanish, Italian, Arabic, etc.), alongside the age range included, whereas in many countries primary school ranges up to around 12 years. since our inclusion criteria focused exclusively on healthy children, studies involving children with chronic diseases were not included possibly overlooking sociodemographic insights presented in broader clinical studies. In terms of strengths, to our knowledge, this is the first systematic review assessing correlates of adherence to the MD among school-age children from Mediterranean countries. Multiple databases were searched and screened by independent researchers (PubMed, Psych INFO, and Web of Science) to gather articles from a variety of publications and disciplines of research. We followed the PRISMA guidelines in the methodology to ensure reaching the required standards for reporting systematic reviews and it was registered in the PROSPERO database. We used a validated tool to perform the quality assessment of the included papers [22]. A formal inter-rater reliability coefficient (e.g., Cohen’s or Fleiss’ kappa) could not be calculated; however, a rigorous consensus process was implemented among the reviewers to ensure consistent and reliable study selection and quality assessment. All included studies reported statistical association using a reliable dietary assessment and scoring tool to quantify adherence to the MD (KIDMED) [60].

In conclusion, this systematic review showed that a limited number of studies with weak to moderate quality examined the correlates of MD adherence in preschool and school-aged children. The included studies showed that interpersonal factors were the most investigated correlates, with a limited assessment of individual and environmental ones. This highlights the need for higher-quality studies identifying further individual and environmental factors of the SEM and their impact on MD adherence. It is further recommended to conduct high-quality studies from the remaining Mediterranean countries on this age group covering the Middle Eastern and African regions. To build on the respective interventions and translate the current findings to practical programs, it is crucial to have a comprehensive understanding of the different levels of correlates and their impact on the children’s MD adherence and consequently, their overall health. Interventions should target children and parents to achieve the desired changes and reinforce MD adherence. Such interventions will not only improve the dietary status of school-age children but will also pave the way toward the achievement of a stronger adherence to MD in adulthood.

## Supplementary Information

Below is the link to the electronic supplementary material.

Supplementary file 1 (PDF 57 kb)

Supplementary file 2 (PDF 174 kb)
