# Supplementary material for: Correlates of adherence to the Mediterranean diet among preschool-age and school-age children living in Mediterranean countries: a systematic review
Source: Eur J Nutr. 2025 Aug 21;64(6):264. doi: 10.1007/s00394-025-03769-9 (PMC12370561; doi:10.1007/s00394-025-03769-9)
Supplement: Supplementary file 1 — (PDF 57 kb) [file 394_2025_3769_MOESM1_ESM.pdf]

Search Strategy

"Mediterranean diet" OR "Mediterranean dietary pattern"<br>2 "Determin*" OR "Caus*" OR "Condition*" OR "Contributing factor" OR "Correlat*" OR "Factor" OR "Factors" OR "Indicators" OR "indicator" OR "indices" OR "index" OR "Influence" OR "Predictor" OR "predictors" OR "variable" OR "Variables"<br><br>"Individual" OR "Interpersonal" OR "Personal factors" OR "Primary School" OR "School-age children" OR "Mother education" OR "Parental Knowledge" OR "Parental education" OR "Parental adherence" OR "Parental Feeding Practices" OR "food habits" OR "Eating practices" OR "Dietary behaviors" OR "Family" OR "Food consumption pattern" OR "Diet aspects" OR "Dietary intakes" OR "Parent* belief*" OR "Parent attitude*" OR "Parent* control" OR "Home environment" OR "household environment"<br><br>"Collective" OR "Socio-cultural" OR "Sociocultural" OR "Socio-economic" OR "Socioeconomic" OR "Sociodemographic" OR "Socio-demographic" OR "Societ*" OR "Environmental factors" OR "ethnic*" OR "cultur*" OR "income" OR "Parent's occupation" OR "Food laws" OR "Food polic*" OR "Food strateg*" OR " Food regulations" OR "Food programs" OR "School meals" OR "Snack programs" OR "School feeding programs" OR "Economic crisis" OR " Economic situation" OR "Food availability" OR "Food access*" OR "Food security" OR "Food sustainability" OR "Culinary" OR "Diet diversity" OR "Geographical Region" OR "School time" OR "School schedule" OR "School environment"
