# Supplementary material for: Correlates of adherence to the Mediterranean diet among preschool-age and school-age children living in Mediterranean countries: a systematic review
Source: Eur J Nutr. 2025 Aug 21;64(6):264. doi: 10.1007/s00394-025-03769-9 (PMC12370561; doi:10.1007/s00394-025-03769-9)
Supplement: Supplementary file 2 — (PDF 174 kb) [file 394_2025_3769_MOESM2_ESM.pdf]

Supplementary Material (S2)- Quality Analysis

**References**	**Part A- Q1** Are the individuals selected to participate in the study likely to be representative of the target population?	**A Q2** What percentage of selected individuals agreed to participate?	**Part A Section Selection bias Rating**	**Part B - Q 1** Section study design: indicate the study design	**B Q2** Was the study described as randomized? If no go to component C	**Part C - Q1** Were there important differences between groups prior to the intervention	**C Q2** If yes, indicate the percentage of relevant confounders that were controlled (either in the design (ex stratification, matching) or analysis?	**Part C Section Confounders Rating**	**Part D - Q1** Was the outcome assessor aware of the intervention or exposure status of participants?	**D Q2** Were the study participants aware of the research question?	**D Section Blinding rating**	**E Q1** Were the data collection tools shown to be valid?	**E Q2** Were data collection tools shown to be reliable?	**PART E Section Data collection methods Rating**	**Part F - Q1** Were withdrawals and drop-outs reported in terms of numbers and/or reasons per group?	**F Q2** Indicate the percentage of participants completing the study (if the percentage differs by groups, record the lowest).	**"PART F Section Withdrawals and drop-outs Rating"**	**"Part G - Q1** Is the tool used for exposure assessment validated?"	**"G Q2** Is the tool used for exposure assessment reliable?"	**"PART G Exposure assessment rating"**	**GLOBAL RATING**
Sotos-Prie	2 Somewhat	5 Can't tell	**2 Mo**	7 Othe	YES **B Q3**	2 no	2 60-79%	**1 strong**	3 Can't tell	1 Yes	**2 Mo**	1 Yes	3 Ca	**2 Mo**	"4 not appl	"5 not appli	**Not Appl**	1 Yes	3 Can	**2 mo**	**1 strong**

sto et al. (2015)	Likely		**derate**	r specify	If yes, was the method of randomization described? **YES B Q4** If yes, was the method appropriate? **YES**		(some)				**derate**		n't tell	**derate**	icable (i.e. one time surveys)"	cable (i.e. retrospective study)"	**icable**		't tell	**derate**	**(no weak ratings)**
Zani et al. (2016)	2 Somewhat Likely	"3 less than 60%	**3 Weak**	"5 cohort	NO	1 yes	3 less than 60% (Few or None)	**3 Weak**	3 Can't tell	1 Yes	**2 Moderate**	1 Yes	3 Can't tell	**2 Moderate**	1 yes	1 80-100 %	**1 Strong**	1 Yes	3 Can't tell	**2 moderate**	**3 weak (two or more weak ratings)**
Buja et al. (2020)	3 Not likely	agreement"	**3 Weak**	(one group	NO	2 no	1 80-100 % (most)	**1 strong**	3 Can't tell	1 Yes	**2 Moderate**	1 Yes	1 Yes	**1 Strong**	"4 not applicable	"5 not applicable	**Not applicable**	1 Yes	1 Yes	**1 Strong**	**2 moderate (on**

				pre + post (before and after))"											(i.e. one time surveys)"	(i.e. retrospective study)"					**e weak rating)**
Costarelli et al. (2021)	3 Not likely	1 80-100% agreement	**3 Weak**	7 Other specify	NO	1 yes	1 80-100% (most)	**1 strong**	1 Yes	1 Yes	**3 Weak**	3 Can't tell	3 Can't tell	**3 Weak**	"4 not applicable (i.e. one time surveys)"	"4 not applicable (i.e. one time surveys)"	**Not applicable**	1 Yes	1 Yes	**1 Strong**	**3 weak (two or more weak ratings)**
Dayi et al. (2021)	3 Not likely	5 Can't tell	**3 Weak**	7 Other specify	NO	1 yes	"3 less than 60% (few or none)"	**3 Weak**	1 Yes	1 Yes	**3 Weak**	1 Yes	1 Yes	**1 Strong**	"4 not applicable (i.e. one time surveys)"	"4 not applicable (i.e. one time surveys)"	**Not applicable**	1 Yes	3 Can't tell	**2 moderate**	**3 weak (two or more weak ratings)**
Grassi et	2 Somewhat	2 60-79%	**2 Mo**	7 Othe	NO	3 can't tell	4 can't tell	**3 Weak**	1 Yes	1 Yes	**3 Weak**	1 Yes	3 Ca	**2 Mo**	"4 not appl	"4 not appli	**Not appli**	3 Can'	3 Can	**3 weak**	**3 weak**

al.(2020)	Likely	agreement	**derate**	r specify									n't tell	**derate**	icable (i.e. one time surveys)"	cable (i.e. one time surveys)"	**cable**	t tell	't tell		**(two or more weak ratings)**
Bučan Nenadić et al. (2021)	2 Somewhat Likely	2 60-79% agreement	**2 Moderate**	7 Other specify	NO	3 can't tell	4 can't tell	**3 Weak**	1 Yes	1 Yes	**3 Weak**	1 Yes	3 Can't tell	**2 Moderate**	"4 not applicable (i.e. one time surveys)"	"4 not applicable (i.e. one time surveys)"	**Not applicable**	3 Can't tell	3 Can't tell	**3 weak**	**3 weak (two or more weak ratings)**
Romanos-Nanclares et al. (2018)	4 Can't tell	5 Can't tell	**3 Weak**	7 Other specify	NO	3 can't tell	4 can't tell	**3 Weak**	1 Yes	1 Yes	**3 Weak**	1 Yes	1 Yes	**1 Strong**	"4 not applicable (i.e. one time surveys)"	"4 not applicable (i.e. one time surveys)"	**Not applicable**	2 no	2 no	**3 weak**	**3 weak (two or more weak ratings)**
Obradovic Salcin et al.	1 Very likely	5 Can't tell	**1 Strong**	7 Other specify	NO	3 can't tell	"3 less than 60% (few or	**3 Weak**	1 Yes	1 Yes	**3 Weak**	1 Yes	1 Yes	**1 Strong**	1 yes	1 80-100 %	**1 Strong**	3 Can't tell	3 Can't tell	**3 weak**	**3 weak (two or more**

(2019)							none)"														**weak ratings)**
Sanlier et al. (2021)	3 Not likely	1 80-100% agreement	**3 Weak**	7 Other specify	NO	2 no	4 can't tell	**3 Weak**	1 Yes	1 Yes	**3 Weak**	1 Yes	1 Yes	**1 Strong**	"4 not applicable (i.e. one time surveys)"	"4 not applicable (i.e. one time surveys)"	**Not applicable**	1 Yes	1 Yes	**1 Strong**	**3 weak (two or more weak ratings)**
Torres-Luque et al. (2018)	4 Can't tell	5 Can't tell	**3 Weak**	7 Other specify	NO	3 can't tell	4 can't tell	**3 Weak**	1 Yes	1 Yes	**3 Weak**	1 Yes	1 Yes	**1 Strong**	"4 not applicable (i.e. one time surveys)"	"4 not applicable (i.e. one time surveys)"	**Not applicable**	1 Yes	1 Yes	**1 Strong**	**3 weak (two or more weak ratings)**
Oliver Olid et al. (2023)	3 Not likely	5 Can't tell	**3 Weak**	7 Other specify	NO	3 can't tell	4 can't tell	**3 Weak**	1 Yes	1 Yes	**3 Weak**	1 Yes	1 Yes	**1 Strong**	2 No	4 can't tell	**Weak**	1 Yes	3 Can't tell	**2 moderate**	**3 weak (two or more weak rati**

																					**ngs)**
